# Apremilast treatment of immune-mediated inflammatory skin diseases: a narrative review

**DOI:** 10.3389/fphar.2025.1633426

**Published:** 2025-08-21

**Authors:** Xiaoqian Liang, Jiecheng Zheng, Xueyi Huang, Min Tan, Jia Liao

**Affiliations:** Department of Dermatology, Zhongshan Second People’s Hospital, Zhongshan, Guangdong, China

**Keywords:** apremilast, phosphodiesterase 4 inhibitor, immune-mediated inflammatory skin disease, cytokines, small-molecule drug

## Abstract

Novel therapeutic approaches on molecular pathways are being developed to treat inflammatory and autoimmune cutaneous dermatoses. Apremilast is an orally administered small-molecule phosphodiesterase 4 (PDE4) inhibitor that upregulates intracellular cyclic 3′,5′-adenosine monophosphate (cAMP) levels to mediate a large array of proinflammatory cytokines as well as exerts its anti-inflammatory functions and therapeutic efficacy in skin diseases rather than an immunosuppressive mode of action. Early-phase clinical trials have demonstrated its favorable efficacy such that the United States Food and Drug Administration (USFDA) has approved its use for the treatment of psoriasis, psoriatic arthritis, and Behçet’s syndrome. Compared to conventional immunosuppressive therapies, apremilast has better safety and tolerability profiles that significantly reduce the risk of serious adverse reactions from long-term usage. Apremilast shows easier and faster absorption even by special areas of the body, such as nails, scalp, palms and soles of feet, and genitals, along with clinically meaningful improvements. More recently, accumulating real-world evidence has revealed that it is highly effective for treating multiple immune-mediated inflammatory skin diseases in an off-label manner; it also appears to be useful either alone or as an add-on treatment against some chronic inflammatory skin disorders recalcitrant to conventional therapies. Thus, further large-scale studies and real-life trials are necessary to better elucidate its role in dermatology. The present narrative review provides an overview of apremilast as a novel therapeutic option for skin disorders, including a comprehensive look at its pharmacology, clinical efficacy, and safety profile, with the aim of enlightening clinicians on the broad applications and full potential of this small-molecule drug based on currently available evidence.

## 1 Introduction

**FIGURE 1:**
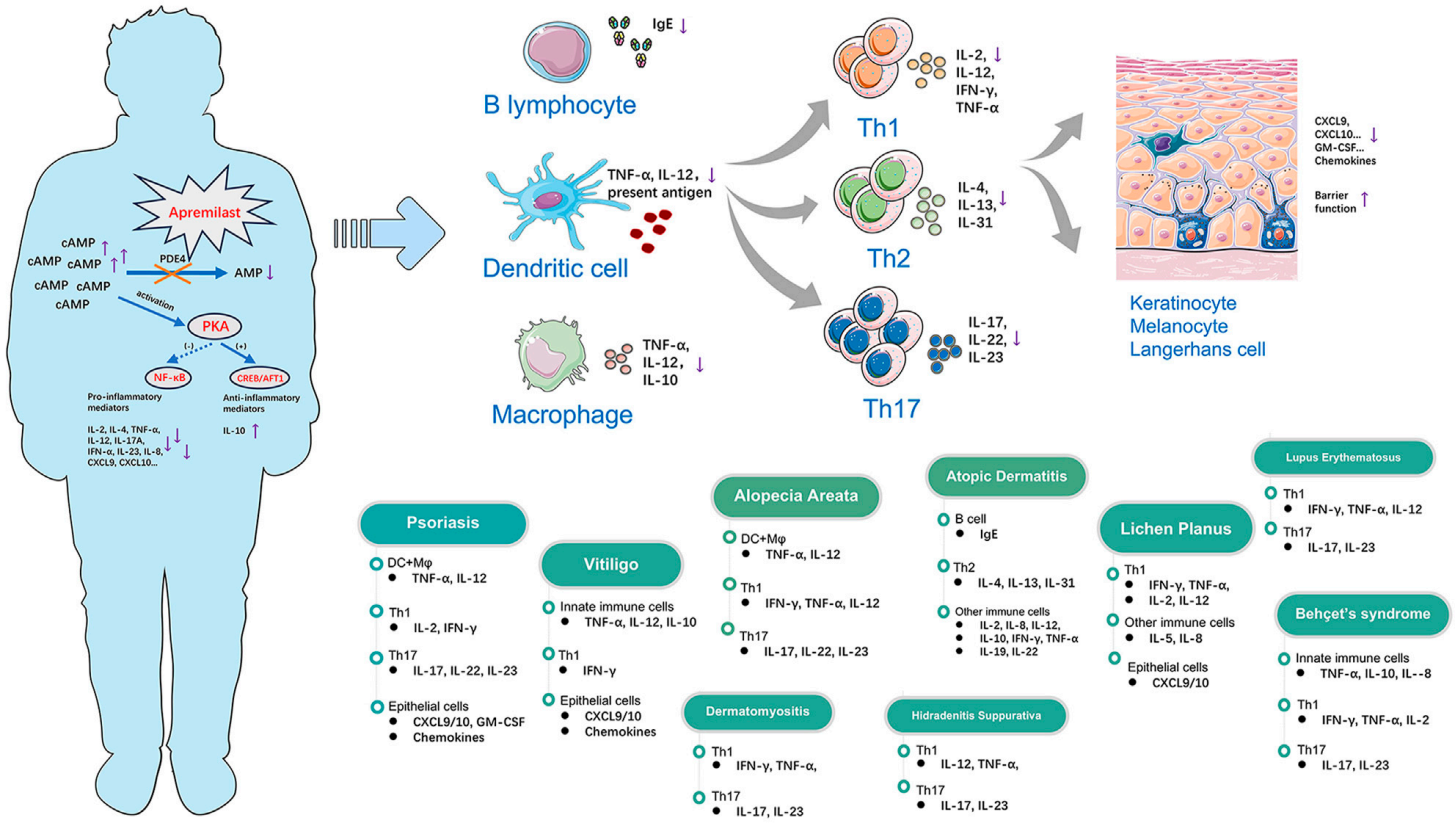
Modes of action of apremilast in the regulation of multiple immune-mediated inflammatory skin diseases through immunocytes and cytokines.

Phosphodiesterase 4 (PDE4) plays a significant role in modulating inflammatory responses by degrading cyclic 3′,5′-adenosine monophosphate (cAMP), which is expressed by various structural cell types. Apremilast is an oral small-molecule PDE4 inhibitor that has shown impressive disease-modifying potency and anti-inflammatory activity in chronic inflammatory dermatoses; hence, it has been approved by the United States Food and Drug Administration (USFDA) for the treatment of moderate-to-severe plaque psoriasis, psoriatic arthritis (PsA), and Behçet’s syndrome in adults. It has been found to accurately inhibit PDE4 isoforms from all four subfamilies (A1A, B1, B2, C1, and D2) as well as selectively regulate the production of inflammatory mediators, including T helper (Th1, Th2, and Th17) cytokines. In T cells and monocytes, apremilast was found to activate phosphorylation of the protein kinase A substrates CREB as well as activate transcription factor-1 (PKA-CREB/ATF-1) pathways while inhibiting NF-κB-driven transcriptional activity, thereby inducing TLR4 signaling dysregulation and reducing type I interferon (IFN)-α production. Unlike immunosuppressors like thalidomide and lenalidomide, apremilast has minimal effects on B-cell differentiation, immunoglobulin and T-cell clonal expansions, as well as *in vivo* antibody responses, suggesting that its impacts on innate immunity are greater than those on adaptive immunity along with better safety profile (Schafer et al., 2014). In dermatology, preliminary studies have highlighted the potential efficacy of apremilast against various immune-mediated inflammatory skin disorders. In the present review, we summarize the existing clinical practices with regard to apremilast application in immune-mediated inflammatory skin diseases (Table 1), including psoriasis, vitiligo, alopecia areata, atopic dermatitis, lichen planus, dermatomyositis, hidradenitis suppurativa, lupus erythematosus, and Behçet’s syndrome, along with a primary focus on its associated action mechanisms (Figure 1; Table 2).

**TABLE 1:** Overview of the key clinical trials involving apremilast for immune-mediated skin diseases.

Trial name (phase)	Disease	Study design (patients, dosage)	Efficacy outcome	Safety profile (AEs ≥5%)	Follow-up	Notes
ESTEEM 1/2 (phase III) (Papp et al., 2015; Paul et al., 2015)	Moderate-to-severe psoriasis	Multicenter, randomized, double-blind, placebo-controlled. N = 1,257 patients were randomized 2:1 to apremilast 30 mg BID or placebo	- PASI 75 at week 16: 33.1% (ESTEEM 1) and 28.8% (ESTEEM 2) in apremilast vs. 5.3% and 5.8% in placebo - NAPSI-50 at week 16: 33.3% (ESTEEM 1) and 44.6% (ESTEEM 2) in apremilast vs. 14.9% and 18.7% in placebo	- Common AEs ESTEEM 1: diarrhea (18.8%), nausea (15.7%), upper respiratory tract infection (10.2%), nasopharyngitis (7.3%), tension headache (7.3%), and headache (5.5%) ESTEEM 2: diarrhea (15.8%), nausea (18.4%), nasopharyngitis (7.4%), tension headache (7.4%), headache (6.3%), and vomiting (5.1%)	104 weeks	- Gastrointestinal AEs resolved within 1 month in most cases - FDA approval for psoriasis based on these trials. - Efficacy maintained in long-term extension studies
LIBERATE (phase IIIB) (Reich et al., 2017)	Moderate-to-severe psoriasis	Randomized, double-blind, placebo-controlled N = 250 patients were randomized 1:1:1 to placebo, apremilast 30 mg BID, or etanercept 50 mg weekly for 16 weeks, then all switched to apremilast	- PASI 75 at week 16: 39.8% (apremilast) vs. 48.2% (etanercept) vs. 11.9% (placebo) - Sustained response: PASI 75 at week 52: 50.6% (apremilast/apremilast), 55.4% (etanercept/apremilast), and 46.4% (placebo/apremilast)	Acceptable and similar to known profiles. No new safety signals were reported in comparison with the ESTEEM program data	52 weeks	At week 16, there were non-significant differences of PASI 75 achievement between apremilast and etanercept; switching from etanercept to apremilast was tolerated well
PALACE 3 (phase III) (Edwards et al., 2016)	PsA with skin involvement	Randomized, double-blind, placebo-controlled. N = 505 patients randomized 1:1:1 to placebo, apremilast 20 mg BID, or 30 mg BID.	- Skin efficacy: PASI 50 achieved by 41% (30 mg) and 24% (placebo) at week 16 - PsA efficacy: ACR20 response rates at week 16: 28% (20 mg) and 41% (30 mg) vs. 18% (placebo)	At week 16, placebo vs. 20 mg vs. 30 mg Diarrhea (2% vs. 15% vs. 16%), nausea (5% vs. 11% vs. 14%), headache (5% vs. 9% vs. 12%), and upper respiratory tract infection (2% vs. 7% vs. 7%).	52 weeks	- Demonstrated dual efficacy in PsA joint symptoms and psoriasis
Vitiligo phase II trial (Khemis et al., 2020)	Non-segmental vitiligo	Randomized, double-blind, placebo-controlled trial N = 77 patients received apremilast + UVB vs. placebo + UVB (1:1)	- VASI score change at week 24: no significant difference between groups	- AEs: consistent with psoriasis trials	52 weeks	-
Vitiligo phase II trial (Sharma et al., 2023)	Progressive non-segmental vitiligo	Randomized, controlled, parallel-group, open-labeled N = 31 patients received add-on apremilast 30 mg BID + standard treatment vs. standard treatment (16:15)	The first indication of repigmentation was observed at week 4 (add-on apremilast group) vs. week 7 (control group)	Diarrhea and weight loss	12 weeks	The VASI score, body mass index (BMI), DLQI, and BMI in the add-on apremilast group reduced significantly
Alopecia areata phase II trial (Mikhaylov et al., 2018)	Moderate-to severe alopecia areata	Randomized, placebo-controlled, single-center pilot N = 30 patients were randomized 1:2 to placebo or apremilast 20 mg BID.	40% (8/20) of the patients in the apremilast arm withdrew mostly due to lack of efficacy and AEs; only 1 of 12 apremilast-treated subjects achieved 50% reduction in SALT50 at 24 weeks, and 1 of 8 placebo-treated subjects achieved SALT50	There are non-significant differences in the AEs between the two groups	48 weeks	-
Alopecia areata case series (Taneja and Gupta, 2020)	Recalcitrant alopecia areata	A retrospective analysis at a tertiary care center in India N = 15 patients were treated with apremilast 30 mg QD or 30 mg BID (11:4)	All patients demonstrated hair regrowth, with four patients (26.7%) showing good responses (75%), nine patients (60%) showing moderate responses (50%–74%), and two patients (13.3%) showing mild improvements (25%–49%)	Gastrointestinal side effects included nausea, vomiting, and diarrhea, which were bothersome in the majority (N = 11, 73.3%) of patients and required reduction of dose to 30 mg QD, with similar efficacy	8 weeks	It indicated that apremilast could be a potential treatment for refractory alopecia areata at a lower dose
Atopic dermatitis phase II trial (Samrao et al., 2012)	Moderate-to-severe atopic dermatitis	Open-label, single-arm N = 16 patients were treated with apremilast 20 mg BID or 30 mg BID (6:10)	Both groups displayed significant reductions of EASI, VAS, and DLQI	Nausea was the most common AE and dose-related. One subject developed herpes zoster	24 weeks	- Limited sample size; no control group
Atopic dermatitis phase II trial (Simpson et al., 2019)	Moderate-to-severe atopic dermatitis	Double-blind, placebo-controlled study N = 185 patients were randomized to placebo, apremilast 30 mg BID, or 40 mg BID (64:58:63)	At week 12, a dose–response relationship was observed in the apremilast 40 mg BID group but not in the 30 mg BID group, which led to statistically significant improvements vs. placebo in EASI score	Apremilast 30 mg BID: consistent with psoriasis trials Apremilast 40 mg BID: AEs were more frequent and cellulitis occurred (N = 6)	24 weeks	Apremilast 40 mg BID showed modest efficacy and decreased AD-related biomarkers in moderate-to-severe AD
Lichen planus phase II trial (Viswanath et al., 2022)	Lichen planus	Single-center, non-randomized, open-label, pilot study N = 26 patients were treated with apremilast 30 mg BID	At week 12, 34.61% (9/26) patients attained improvements of two or more grades as per PGA, and 42.30% (11/26) patients achieved more than 50% improvements	23.07% (6/26) patients developed one or more AEs: headache (N = 4, 15.38%), diarrhea (N = 3, 11.53%), and abdominal pain (N = 1, 3.84%)	12 weeks	-
Dermatomyositis phase II trial (Bitar et al., 2022)	Cutaneous dermatomyositis	Open-label, single-arm, non-randomized controlled study N = 8 patients were treated with apremilast 30 mg BID as add-on treatment	An overall response rate of 87.5% was found at 3 months among seven patients	Well tolerated, with no grade 3 or higher AEs	24 weeks	Associations with downregulation of multiple inflammatory pathways and cytokines were found
Hidradenitis suppurativa phase II trial (Vossen et al., 2019b)	Moderate hidradenitis suppurativa	Randomized, double-blind, placebo-controlled study N = 20 patients were randomized 1:3 to placebo and apremilast 30 mg BID.	- Clinical response at week 16: 53.3% (apremilast) vs. 0% (placebo, *p* = 0.055) - Abscess/nodule count: mean reduction of 2.6 - Long-term follow-up: 50% of the responders maintained responses at 2 years	Headache and nausea; no discontinuations due to AEs	52 weeks (compassionate use extension)	- Small sample size limits conclusions; phase III trials pending
Discoid lupus erythematosus phase II trial (De Souza et al., 2012)	Discoid lupus erythematosus	Open-label, single-arm, pilot study N = 8 patients were treated with apremilast 20 mg BID	Significant reduction in the CLASI and cutaneous lupus erythematosus activity at day 85	Nausea, headache, and diarrhea; mild and transient	12 weeks	-
Behçet’s syndrome phase II trial (Vieira et al., 2020)	Refractory Behçet’s syndrome	French nationwide multicenter observation study N = 50 patients were treated with apremilast 30 mg BID	Overall response rates of 74% for the entire cohort and 70% for the mucocutaneous-articular cluster were observed	Approximately 33 (66%) patients experienced AEs of mostly diarrhea (38%), nausea (34%), and headache (32%)	11 months	Findings shed light on the effectiveness and tolerability of apremilast in BS patients with refractory joint and mucocutaneous manifestations

AE, adverse events; PASI, psoriasis area and severity index; NAPSI, nail psoriasis severity index; VASI, vitiligo area scoring index; DLQI, dermatology life quality index; EASI, eczema area and severity index; VAS, visual analog scale; PGA, physician global assessment; CLASI, cutaneous lupus erythematosus disease area and severity index.

**TABLE 2:** Cytokines targeted by apremilast in disease-specific signaling pathways.

Disease	Key immunocytes targeted	Key cytokines targeted	Outcome
Plaque psoriasis	Th1, Th17, DC, and Mφ	IL-2, IFN-γ, IL-17, IL-22, IL-23, TNF-α, and IL-12	Effective
Psoriatic arthritis	Th1 and DC	IL-2, IL-4, TNF-α, IL-12, and IL-17A	Effective
Vitiligo	Th1	IFN-γ and TNF-α	Controversial
Alopecia areata	Th1, Th17, DC, and Mφ	IFN-γ, TNF-α, IL-12, IL-17, IL-22, and IL-23	Controversial
Atopic dermatitis	Th2	IL-4, IL-13, and IL-31	Effective
Lichen planus	Th1	IFN-γ, TNF-α, IL-2, and IL-12	Effective
Dermatomyositis	Th1 and Th17	IFN-γ, TNF-α, IL-17, and IL-23	Effective
Hidradenitis suppurativa	Th1 and Th17	IL-12, TNF-α, IL-17, and IL-23	Modest/limited efficacy
Lupus erythematosus	Th1 and Th17	IFN-γ, TNF-α, IL-12, IL-17, and IL-23	Modest/limited efficacy
Behçet’s syndrome	Th1, Th17	IFN-γ, TNF-α, IL-12, IL-17, and IL-23	Effective

Th, T helper; DC, dendritic cell; Mφ, macrophage; IL, interleukin; IFN, interferon; TNF, tumor necrosis factor.

## 2 Apremilast in immune-mediated inflammatory skin diseases

### 2.1 Psoriasis and PsA

Psoriasis is a multifactorial chronic inflammatory skin disease that affects approximately 2%–4% of the global population (Parisi et al., 2013); it is associated with several comorbidities, including PsA, with a concurrency rate of over 20% statistically that can seriously harm the physical and mental health of patients. Extant studies have highlighted the critical interplay between Th17-related cells and cytokine cascades in the pathogenesis of psoriasis and PsA. It is well acknowledged that dendritic cells release IL-12, IL-23, and other cytokines to expand the differentiated Th17 cell populations; therefore, the Th1, Th22, and Th17 subsets produce proinflammatory mediators, including IL-17A/F, tumor necrosis factor (TNF)-α, IFN-α, and IL-22, which result in keratinocyte hyperproliferation and immunocyte infiltration in psoriatic skin (Girolomoni et al., 2017). Similarly, in PsA, upregulated expression of TLR2 in the dendritic cells can induce Th1 responses and release proinflammatory cytokines like IL-2, IL-4, TNF-α, IL-12, and IL-17A, thereby triggering inflammatory responses (Ocampo and Gladman, 2019).

Although the innovative biologics available in the market have shown great efficacy in alleviating psoriasis and PsA, they are prone to limitations like primary and secondary failures, safety concerns, and unaffordable costs resulting in treatment discontinuation. *In vivo*, PDE4 is expressed in various structural cell types in both keratinocytes and the synovium. Along with inhibition of the PDE4 enzyme, apremilast deregulates production of substantial chemokines and cytokines, including CXCL9, CXCL10, TNF-α, IFN-α, IL-2, IL-12, and IL-23, in the human primary peripheral blood mononuclear cells as well as TNF-α in the natural killer cells and epidermal keratinocytes, as suggested in a preclinical model of psoriasis. Moreover, apremilast increases the expression of the anti-inflammatory mediator IL-10 as well as reduces human leukocyte antigen-DR and intercellular cell adhesion molecule-1, thus displaying a broad range of anti-inflammatory activities (Schafer et al., 2010).

Clinical experience with apremilast has demonstrated its potential to alter the landscape of small-molecule immunotherapy of psoriasis. Additionally, it has yielded favorable efficacies at difficult-to-treat sites, such as the scalp, face, nails, and genitals, as well as against palmoplantar pustulosis. As a small-molecule drug, apremilast shows more effective and faster onset in the small blood vessels of the palmoplantar areas as well as demonstrates comparable efficacy to methotrexate; however, there is notable heterogeneity in the outcome measures and risk of bias across studies, which highlights the need for standardized evaluation metrics (Spencer et al., 2023). In the clinic, it is likely employed as a maintenance therapy following conventional treatment; circumstantially, it is even used as a post-biologic or included in combination with other biologics. Additionally, apremilast has been shown to be an alternative approach for PsA patients who failed traditional disease-modifying antirheumatic drugs (DMARDs) for their limited efficacy and considerable toxicity (Sandhu et al., 2020). The long-term efficacy and safety of apremilast in psoriasis and PsA have been evaluated in several large-scale clinical trials, even though its overall effectiveness appears to be less than those of biologic agents (Torres and Puig, 2017). For patients having underlying active or latent infectious diseases, including tuberculosis and viral hepatitis, along with hepatic or renal insufficiency, apremilast is the preferred option. Furthermore, apremilast has been demonstrated to improve the quality of life and have a high safety profile in psoriatic patients with malignancies (Tsentemeidou et al., 2022; Podevin et al., 2023). However, concerns on whether apremilast could inhibit structural joint damage remain undetermined and need to be explored in future clinical trials.

### 2.2 Vitiligo

Vitiligo is an autoimmune disorder that affects pigmentation and is prevalent in approximately 0.5%–2% of the global population (Bergqvist and Ezzedine, 2020). It is well acknowledged that IFN-γ signaling and C-X-C motif chemokine ligand 9/10 axis contribute to the primary pathogenesis of vitiligo. IFN-γ signals the Langerhans cells and keratinocytes in the epidermis, thereby stimulating the production of chemokines CXCL9/10 in the dermis that are subsequently recognized by CXCR3, resulting in the recruitment of effector CD8^+^ T cells. The autoreactive T cells induce initial apoptosis of the melanocytes to establish long-lived CD8^+^ resident memory T cells within the skin (Frisoli and Harris, 2017; Richmond et al., 2017). It has been reported that the CD8^+^ resident memory T cells could possess cytotoxic effector functions when exposed to inflammatory cytokines and are therefore functionally associated with the disease maintenance of vitiligo (Cheuk et al., 2017). Current therapeutics that suppress autoimmune inflammation could help reverse repigmentation of the skin. By elevating the intracellular cAMP and anti-inflammatory cytokine IL-10 levels, apremilast decreases the production of CXCL9/10 by inhibiting IFN-γ signaling. A case series reported by Majid et al. (2019) showed the positive outcomes of oral apremilast in the disease control of 13 adult patients with onset vitiligo; however, this study lacked a control or comparison group as well as a sufficiently large population size.

A systematic review of apremilast involving 122 participants demonstrated its significant efficacy for the treatment of vitiligo. Furthermore, experimental studies have suggested that the combination of apremilast and narrowband ultraviolet B (UVB) phototherapy is more effective in promoting repigmentation supported by significant decreases in CD8^+^ T cells, CD11c^+^ dendritic cells, and Th17-related markers along with upregulation of melanogenesis markers (Kim et al., 2023). Sharma et al. (2023) reported that 30 mg of add-on apremilast twice daily in combination with standard treatment could reduce disease progression and accelerate clinical improvement of vitiligo. However, in a double-blind and placebo-controlled study, 77 patients were randomized 1:1 to apremilast in combination therapy with UVB and placebo with UVB, which showed no significant difference between the groups at week 24 (Sharma et al., 2025). It was suspected that such varying clinical efficacies could be due to a combination of patient-specific factors, including anatomical location, disease stage (progressive/quiescent), variability in immune profiles, and variations in PDE4 subtype expressions; the small sample size, short follow-up, and similar demographic characteristics may also have limited the conclusions. Nevertheless, the results of multiple trials have shown that apremilast is still effective and has anti-inflammatory efficacy in the treatment of vitiligo.

### 2.3 Alopecia areata

Alopecia areata (AA) is a hair loss disorder the affects approximately 2% of the general population and is characterized by cytokine dysregulation of IFN-γ, IL-15, IL-2, and IL-7 that mediate CD8^+^ T-cell proliferation, resulting in immune-privilege collapse of the hair follicle. Recent research has shown that PDE4-related cytokine pathways are activated and that PDE4 is upregulated in the scalp lesions in AA patients (Suárez-Fariñas et al., 2015). In animal models, hair regrowth was observed following PDE4 antagonism. Humanized AA mice treated with apremilast showed almost complete absence of CD8^+^ T cells and decreased levels of proinflammatory cytokines, including IFN-γ and TNF-α, despite the relatively low number of experimental mice owing to difficulties with human scalp transplants (Laufer Britva et al., 2020; Keren et al., 2015). Furthermore, there are cases demonstrating significant improvements (Estebanez et al., 2019; Magdaleno-Tapial et al., 2019). A retrospective analysis involving 15 AA patients showed that apremilast was effective against recalcitrant AA over a follow-up period of 1.5 years (Taneja and Gupta, 2020). Intriguingly, Chhabra and Verma (2019) reported a case of steroid-resistant pediatric alopecia totalis responding to apremilast (30 mg/d) and platelet-rich plasma as an adjuvant, indicating their synergistic effects. Mikhaylov et al. (2018) conducted a randomized placebo-controlled study, in which 40% (8/20) of the patients in the apremilast arm withdrew mostly owing to lack of efficacy and adverse events; here, only one of the 12 apremilast-treated subjects achieved 50% reduction in the severity of alopecia tool (SALT) score (SALT50) at 24 weeks, and only one of the eight placebo-treated subjects achieved SALT50, indicating that apremilast may be ineffective for moderate-to-severe AA (Mikhaylov et al., 2018). Some other similar small-scale clinical studies have shown unsatisfactory results for apremilast treatment efficacy in AA patients, indicating that apremilast is less likely to be effective in severe and recalcitrant AA (Weber et al., 2020; Sakakibara et al., 2019; Liu and King, 2017; Gupta et al., 2023; Rudnicka and Olszewska, 2024). The non-responsiveness in these aforementioned studies may be attributed to the small sample sizes, short durations of therapy, and the fact that most patients had alopecia universalis. Additionally, it is speculated that apremilast is only effective in AA patients with significant PDE4 overexpression. Still, the role of apremilast in refractory AA warrants further evaluation, and the reasons for heterogeneous therapeutic responses in the clinic need to be further delineated in upcoming clinical trials.

### 2.4 Atopic dermatitis (AD)

AD is also known as atopic eczema and is a chronic recurrent inflammatory skin disorder characterized by xerotic skin and acute flare-ups of highly pruritic eczematous lesions that affects approximately 2%–4% of adults and up to 20% of children globally (Barbarot et al., 2018; Silverberg et al., 2021). The pathogenesis of AD involves breakdown of the skin barrier due to Th1/Th2 imbalance with cytokine dysregulation, increased immunoglobulin E and eosinophilia through the release of IL-4/5/13, and decreased protection against infection through the release of IL-10. It has been reported that the PDE4 isoforms are overexpressed by up to threefold in the epidermis in AD compared to healthy skin. In murine models, it was observed that AD-associated inflammatory markers were significantly modulated by apremilast (Schafer et al., 2019); the substantial proinflammatory cytokines involved in AD (TNF-α, IL-12, IL-2, IFN-γ, IL-5, and IL-8) were downregulated while the production of anti-inflammatory factors (IL-10) increased. Growing evidence has confirmed the effectiveness and safety of apremilast in the treatment of adults and children with severe AD (Deleanu and Nedelea, 2019; Abrouk et al., 2017). Samrao et al. (2012) conducted an open-label pilot study involving 16 subjects with moderate-to-severe AD; they observed a trend toward improvement even in cohorts who received 20 mg of apremilast twice daily. Farahnik et al. (2017) reported the case of a 55-year-old male patient with a lifelong history of AD recalcitrant to topical steroids and cyclosporine who showed improvements in pruritus and erythema following 10 weeks of treatment with apremilast.

Simpson et al. (2019) conducted a phase II, double-blind, and placebo-controlled trial in 185 adult patients; they observed a dose–response relationship between 40 mg of apremilast administered twice daily and a significant remission in moderate-to-severe AD, with gene array analysis displaying reduced levels of the Th17/Th22-associated biomarkers, including IL-19, IL-22, and S100A, in AD lesions. In another group that was administered 30 mg of apremilast BID, statistically significant improvements were not observed in the EASI score compared to placebo (Simpson et al., 2019). Saporito and Cohen (2016) described the case of an 8-year-old boy with refractory AD who was treated with 30 mg of apremilast daily; here, the boy showed quick improvements of pruritus and inflammation within 2 weeks, indicating that apremilast is a safe and effective medication for the pediatric population. Notably, dupilumab is the first USFDA-approved targeted therapy for AD that blocks IL-4 and IL-13 cytokine signaling (Langan et al., 2020). In patients showing inadequate responses to dupilumab, concomitant apremilast could be promisingly applied for enhanced benefits (Cunningham et al., 2023). In patients at risk of malignancy, apremilast was noted to have a safer profile than JAK inhibitors. Apremilast also shows rapid and complete responses in chronic hand eczema with hepatogenic pruritus or hyperkeratotic hand and foot dermatitis (Navarro-Trivino et al., 2019; Bhat et al., 2024). As novel molecular pathways underlying AD are being uncovered, there are advances in disease management and endotype-based targeted therapies. PDE4 activity is notably increased in AD patients, resulting in intracellular cAMP degradation as well as subsequent increased production of proinflammatory cytokines. Although preclinical studies have lacked active comparators like biologics or standard-of-care therapies that have made it difficult to position apremilast relative to the alternatives, apremilast is undoubtedly a promising targeted therapeutic agent for mild-to-moderate AD in both children and adults.

### 2.5 Lichen planus (LP)

LP is a chronic multifactorial inflammatory disease with a heterogeneous clinical presentation that predominantly causes severe pruritus and pain in the skin, nails, and mucous membranes; this condition affects approximately 15%–20% of patients with clinical relapse (Tekin et al., 2024). The pathogenesis of LP has been incompletely investigated but is mainly known to involve T-cell-mediated damage of the basal keratinocytes in lesions by various inflammatory cytokines and chemokines, such as TNF-α, IFN-γ, IL-9, IL-17, IL-22, IL-23, CXCL9, and CXCL10. Regulatory T cells have been detected in the blood of LP patients, indicating that the inflammation is not restricted to the skin (Domingues et al., 2016). Apremilast inhibits the enzyme PDE4 and promotes intracellular cAMP to inhibit proinflammatory cytokine transcription and immunocyte chemotaxis, which ultimately decreases the production of inflammatory mediators like TNF-α, IFN-γ, IL-2, IL-5, IL-8, and IL-12. Therefore, it is plausible that apremilast may be effective against LP. Paul et al. (2013) conducted an open-label pilot study of apremilast for the treatment of moderate-to-severe LP; they describe that three out of 10 patients showed improvements of two or more grades in the physician global assessment (PGA) following 12 weeks of apremilast administration at 20 mg twice daily and that all individuals showed statistically significant clinical improvements. Similarly, Viswanath et al. (2022) reported that 34.61% (9/26) of the patients in their study who were prescribed 30 mg of apremilast twice daily attained two or more grades of improvement as per the PGA, while 42.30% (11/26) of the patients achieved more than 50% improvements in the lesions by week 12; the consistent conclusion here is that apremilast is effective and safe in the management of LP (Viswanath et al., 2022). For small sample sizes and studies lacking a control group, it is difficult to generalize the above results convincingly. For oral lichen planus (OLP) that has been reported to have a malignant transformation rate of 2.3% to oral squamous cell carcinoma (Gonzalez-Moles et al., 2021), apremilast is likely a viable option in difficult-to-treat cases (Perschy et al., 2022; Kim-Lim and Thomas, 2023). An earlier report noted that a Th17 phenotype-associated cytokine profile was detected in erosive OLP, while a skew toward the Th2 and Treg phenotypes was found in reticular OLP (Wang et al., 2016; Piccinni et al., 2014). With regard to recalcitrant erosive OLP, oral apremilast at a dose of 30 mg twice daily showed significant improvement in the disease activity (AbuHilal et al., 2016). In the case series reported by Sil et al. (2023) and Bettencourt (2016), subjects with refractory OLP who received apremilast showed significant improvements at 12-week follow-up. A randomized, double-blind, placebo-controlled trial for genital erosive lichen planus based on 42 adult women showed credible evidence for the use of apremilast in such a burdensome genital dermatosis (Skullerud et al., 2021). Nonetheless, there are very little data available for apremilast as a treatment for nail lichen planus. Given its desirable responses in nail psoriasis, it would be an interesting and essential research direction to investigate the activity of apremilast on nail lichen planus in the future.

### 2.6 Dermatomyositis (DM)

DM is an idiopathic inflammatory myopathy characterized by cutaneous abnormalities that are frequently complicated by interstitial lung disease, which is refractory and recurrent even following treatment with immunosuppressants. Extant studies have revealed the critical roles of the Th1/Th2 pathways in DM, including TNF-α, IFN-γ, IL-4, IL-6, IL-17, IL-18, IL-23, and STAT signaling that have been found to be downregulated with disease improvement (Moneta et al., 2019; Thompson et al., 2018; Giris et al., 2017). In this regard, different case series and small-scale prospective trials have been reported on the off-label use of apremilast in treating DM. A phase IIa, non-randomized, controlled trial of apremilast in eight patients with recalcitrant DM showed that the overall response rate was 87.5% (Bitar et al., 2022); however, the single-arm single-center setting and short intervention course (12 weeks) were the main limitations of this study. Bitar et al. (2019) also reported on three patients with refractory DM who showed 85% improvement in cutaneous DM activity and severity index (CDASI) at 12-week follow-up. In patients with scalp DM, sustained resolution of the scalp pruritus was observed (Kolla et al., 2021; Charlton et al., 2021). In patients with paraneoplastic DM undergoing chemotherapy, immunosuppressants are constantly contraindicated for increased risk of infection, which gives apremilast a distinct advantage (Cabas et al., 2023). Furthermore, emerging evidence has shown that apremilast is a safe and efficient add-on treatment in recalcitrant DM (Elhage et al., 2022; Konishi et al., 2022).

### 2.7 Hidradenitis suppurativa (HS)

HS is a chronic immune-mediated inflammatory follicular occlusive disease that manifests as inflammatory nodules and abscesses to the formation of sinus tracts and scarring; it is closely associated with various comorbidities, including obesity, hypertension, dyslipidemia, and diabetes mellitus. Its immune dysregulation is predominantly mediated by the TNF-α, IL-1β, IL-10, IL-23/Th17, and IL-12/Th1 pathways. TNF-α inhibitors have been demonstrated to aid with remarkable recovery and ongoing remission in the disease control of HS (Kelly et al., 2015; Moran et al., 2017). In a double-blind, randomized, placebo-controlled trial involving 20 subjects with moderate HS, approximately 53.3% (8/15) of the apremilast-treated patients attained positive clinical responses with significantly lower abscess and nodule counts by 16 weeks (Vossen et al., 2019b); furthermore, prolonged efficacy of the treatment was observed after 2 years (Aarts et al., 2021). In another open-label, phase II trial involving 20 adults with mild-to-moderate HS, 65% of the participants showed significant improvements of HS disease activity (Kerdel et al., 2019); similarly impressive results were also reported for other case series (Weber et al., 2017; Garbayo-Salmons et al., 2021). Paradoxically, Vossen et al. (2019a) found that the inflammatory markers in HS lesions treated with apremilast could not be detected with statistically significant changes, whereas elevated S100A12 and IL-17A levels were found to decrease. Given the efficacy of apremilast in the treatment of psoriasis, HS cases with concomitant psoriasis were successfully treated with the off-label use of apremilast in clinics, even as the molecular crosstalk between HS and psoriasis is actively being studied (Proietti et al., 2020; Lanna et al., 2019). In addition, Garcovich et al. (2020) reported the findings in two apremilast-treated adults having severe HS presenting with concomitant PsA who entered remission successfully. For patients with concomitant HS and Crohn’s disease, the combination of guselkumab and apremilast simultaneously showed clinically validated efficacy (Agud-Dios et al., 2022). The abovementioned small and short-term studies underscore the need for larger and long-term trials to clarify the utility of apremilast in HS therapy.

### 2.8 Lupus erythematosus (LE)

Apremilast may constitute a safe and effective therapeutic modality for LE. An open-label study reported the use of 20 mg of apremilast twice daily for treating eight patients with discoid LE and noted a significant (*p* < 0.05) reduction in the chronic LE disease areas and severity index (CLASI) (De Souza et al., 2012). Further observational studies with larger numbers of patients or varying doses of apremilast for treating LE are necessary to verify this pilot open-label study. Moreover, a 44-year-old Japanese woman who developed psoriasis with systemic LE was treated with 30 mg of oral apremilast twice daily and showed improved outcomes with decreased PASI score and systemic LE activity gradually. The pathogenic mechanisms about the Th17 axis have previously been reported together in psoriasis and systemic LE; here, apremilast blocks the production of INF-γ, IL-12, IL-23, and TNF-α, thus suppressing the Th1/Th17-mediated immune responses and exerting anti-inflammatory activity (Ichiyama et al., 2019).

### 2.9 Behçet’s syndrome (BS)

BS is a chronic recurrent inflammatory skin disease characterized by relapsing mucocutaneous and joint disorders with oral ulcers as the cardinal feature. Here, colchicine or systemic glucocorticoids are the first-line therapies that still show unsatisfactory clinical outcomes. Despite the incompletely investigated action mechanisms of apremilast, its therapeutic benefits in BS are believed to be exerted through modulation of the serum cytokine levels, including those of TNF-α, IL-23, IL-2, IL-8, IL-10, IL-17, and IFN-γ (Wakiya et al., 2022; Fukui et al., 2022). Apremilast could strongly inhibit the activation of surface markers, NETosis, as well as reactive oxygen species production on the neutrophils in BS, resulting in the modulation of innate immunity, intracellular signaling, and chemotaxis (Le Joncour et al., 2023). In the clinic, apremilast has demonstrated efficacies in the refractory mucocutaneous and joint manifestations of BS. In a French nationwide multicenter study involving 50 BS patients, apremilast was administered at 30 mg twice a day with overall response rates of 74% for the entire cohort and 70% for the mucocutaneous-articular cluster; however, the continuation of concomitant therapy with colchicine or DMARDs could represent potential confounders to the efficacy evaluations of this study (Vieira et al., 2020). Additionally, a phase III RELIEF study confirmed significant improvements to the quality of life of the patients (Hatemi et al., 2022), consistent with the findings of preliminary real-world observational studies and randomized placebo-controlled trials (De Luca et al., 2020; Hirahara et al., 2021; Iizuka et al., 2022; Takeno et al., 2022; Lopalco et al., 2019; Ozdede and Hatemi, 2021; Atienza-Mateo et al., 2020). The benefits were also found to be sustained for up to 64 weeks with continued treatment and a known safety profile (Hatemi et al., 2021). For patients with milder disease phenotypes of BS, apremilast may be considered a higher priority than TNF inhibitors (Lopalco et al., 2025).

### 2.10 Others

Owing to the wide anti-inflammatory effects of apremilast in regulating the immune system with a positive safety record, it has been used in the management of a sufficient number of inflammatory skin disorders in an off-label manner, including pityriasis rubra pilaris (Krase et al., 2016), granuloma annulare (Joshi and Tschen, 2023), autoimmune blistering diseases like pemphigus (Sigmund et al., 2023) and linear IgA bullous disease (Maione et al., 2023), morphea (Higuchi et al., 2023), chronic actinic dermatitis (Kaushik et al., 2020a), sarcoidosis (Baughman et al., 2012), Darier disease (Kleidona et al., 2024), dissecting cellulitis (Bernard et al., 2023), orofacial granulomatosis (Kaushik et al., 2020b), papuloerythroderma (Yin et al., 2023), perforating dermatosis (McMichael and Stoff, 2021), prurigo nodularis (Todberg et al., 2020), pyoderma gangrenosum (Bordeaux et al., 2022), SAPHO syndrome (Adamo et al., 2018), seborrheic dermatitis (Cohen et al., 2020), chronic graft-versus-host disease (Sobral-Costas et al., 2025), Sjögren’s syndrome (Manfrè et al., 2023), and Sneddon–Wilkinson disease (Magdaleno-Tapial et al., 2020). Theoretically, apremilast could be a promising therapy for diseases with PDE4 upregulation, including rare diseases with shared inflammatory pathways. Apremilast also hints at the possibility of a therapeutic window based on small-molecular pathways for multiple immune-mediated inflammatory skin disorders. In recognizing that the action mechanisms of apremilast remain incompletely characterized, large-scale preclinical studies and real-life trials are necessitated to better elucidate the application of apremilast to autoimmune and inflammatory skin disorders, which would be critical to realizing the full potential of this novel treatment approach.

## 3 Discussion

Apremilast inhibits the enzyme PDE4 to reduce the levels of proinflammatory cytokines and exert its immunomodulatory effects across various diseases in an off-label manner (sometimes) in dermatologic practice. The assessment of its long-term safety is a crucial consideration for extended practical applications. The current guidelines recommend apremilast with no need for laboratory monitoring, but the long-term data on risks remain insufficient. Nausea, vomiting, diarrhea, and gastrointestinal intolerance are some of the commonly observed side effects. Caution should be exercised at the start of treatment for underweight patients, whose body weights should be monitored regularly. Furthermore, long-term safety issues like infections or malignancies may require extended follow-up periods. There are numerous studies on apremilast as a treatment for immune-mediated inflammatory skin diseases; however, the small sample sizes, short follow-up periods, high dropout rates, and/or lack of comparator drugs limit the conclusions of such studies. Data on the responses of specific subgroups (e.g., patients with comorbidities, elderly patients) as well as cumulative safety risks are lacking. Some of the possible study limitations include publication bias (positive results being more likely to be published) and heterogeneity across studies, along with complicated meta-analyses. These contradictory findings may stem from differences in the study design, patient populations, patient heterogeneities, or treatment durations. The variable response rates observed in these studies may be attributed to differences in the genetic polymorphisms, disease severity, or patient adherence. Apremilast works by inhibiting PDE4, which could help explain the variability in the response rates as different diseases or patients exhibit varying PDE4 expression or activities. Gaps in the evidence include findings from pediatric populations, people with specific comorbidities, or the use of concomitant medications. Additionally, most of the reported trials focus on short-term efficacy while lacking long-term safety and efficacy data. Although some extension studies have suggested sustained responses, real-world data with follow-up periods exceeding 5 years are sparse. Addressing these limitations would help identify critical evidence gaps and suggest directions for future research. A critical knowledge gap still remains regarding the role of apremilast in immune-mediated skin diseases; hence, clinical practice guidelines or expert consensus should be formulated to determine the conditions under which apremilast should be recommended.

## 4 Conclusion

Overall, the efficacy and safety of apremilast in dermatological therapies are supported by clinical evidence; recently, apremilast has been applied in the treatment of various immune-mediated inflammatory skin diseases in an off-label manner. However, it remains to be determined whether this small-molecule PDE4 inhibitor could achieve durable long-term responses, for which future clinical trials are still under development. It is anticipated that apremilast deserves greater value and may have more promising prospects in clinical applications.

## 5 Future directions

The broader roles of apremilast in immune-mediated skin diseases are still under investigation. The extant trials on its efficacy and safety are underpowered, with promising yet inconclusive results. Addressing the limitations of these apremilast studies requires focusing on extended sample sizes, methodological rigor, extended follow-up durations, and better alignment with real-world clinical needs. It is worth noting that although apremilast acts by inhibiting PDE4, the understanding of its interactions with disease-specific pathways remains incomplete. This means that mechanistic drivers including the baseline PDE4 expression or cytokine profiles of variable responses across diseases should be investigated. By combining larger, longer, and more diverse trials with standardized endpoints, mechanistic research, and real-world validation, apremilast could be applied in a more personalized and effective manner to immune-mediated skin conditions.
